# Smoking by altering the peri-implant microbial community structure compromises the responsiveness to treatment

**DOI:** 10.3389/fcimb.2022.1040765

**Published:** 2022-10-14

**Authors:** Yuchen Zhang, Sadia Ambreen Niazi, Yuguang Yang, Yiqing Wang, Xiao Cao, Yibing Liu, Yinhu Li, Qin Zhou

**Affiliations:** ^1^ Key Laboratory of Shaanxi Province for Craniofacial Precision Medicine Research, College of Stomatology, Xi’an Jiaotong University, Xi’an, China; ^2^ Centre of Oral Clinical and Translational Sciences, Faculty of Dentistry, Oral and Craniofacial Sciences, King’s College London, London, United Kingdom; ^3^ Department of Advanced Manufacturing and Robotics, College of Engineering, Peking University, Beijing, China; ^4^ Department of Prosthodontics, School and Hospital of Stomatology, Peking University, Beijing, China; ^5^ Shenzhen-Hong Kong Institute of Brain Science-Shenzhen Fundamental Research Institutions, The Brain Cognition and Brain Disease Institute, Shenzhen Institutes of Advanced Technology, Chinese Academy of Sciences, Shenzhen, China

**Keywords:** oral microbiome, smoking, metagenomic sequencing, community structure, local stability, dental implant

## Abstract

Smoking is an essential risk factor for peri-implant diseases. It also hampers the clinical outcomes of peri-implant therapies. Nonetheless, the effect of smoking can go undetected until the emergence of clinical signs. Bacterial-induced inflammation is responsible for the initiation and progression of peri-implant diseases. We hypothesize that smoking impacts the peri-implant microbiome even in status of clinical health, putting it into a sub-healthy condition that responds poorly to peri-implant treatments. To validate this, peri-implant plaque samples from 18 participants including 10 smokers (S) and 8 non-smokers (NS), who had received implant prostheses were analyzed using metagenomic shotgun sequencing. The results showed that in addition to taxonomical and functional differences, the local stability in the S group was also shown to be much higher than that in the NS group, indicating greater stubbornness of the peri-implant microbiome associated with smoking. Besides, the topological structures were also distinct between the two groups. The highly connected species interacted more preferentially with each other in the S group (eigenvector centralization, 0.0273 in S and 0.0183 in NS), resulting in a greater tendency of forming small-world modules (modularity, 0.714 in S and 0.582 in NS). While in the NS group, inter-species correlations were more evenly distributed (clustering coefficient, 0.532 in S and 0.666 in NS). These alterations overall explained the greater stubbornness of the peri-implant microbiome associated with smoking, which may cause poor responsiveness to peri-implant therapies. From a microbial perspective, this may be a potential reason why smoking impacts negatively on the outcome of peri-implant treatments.

## Introduction

Since the conference of Osseointegration in Clinical Dentistry in 1982, dental implants have improved extensively as shown in both laboratory investigations and clinical practice. Implant therapies have evolved into a highly predictable option for treating fully or partially edentulous ridges as they surpass traditional prostheses with significant functional and biological advantages (Buser et al., 2017). Patient-reported outcome has demonstrated that dental implants can achieve a high degree of satisfaction in terms of aesthetics and masticatory functions (Feine et al., 2018), which has made them more acceptable replacement option.

Despite the success of dental implants, some complications must be considered, among which peri-implant diseases is the most prevalent one (Karlsson et al., 2020). Peri-implant diseases usually refer to peri-implant mucositis and its subsequent state peri-implantitis. Peri-implant mucositis is defined as an inflammatory lesion of the mucosa surrounding the implant without influencing the supporting bone matrix (Heitz-Mayfield and Salvi, 2018). In contrast, peri-implantitis is when the inflammation has further caused progressive loss of the supporting bone (Schwarz et al., 2018). According to a meta-analysis conducted in 2017 (Lee et al., 2017), the weighted mean prevalence of peri-implant mucositis and peri-implantitis at the patient level is 46.83% and 19.83%, respectively, highlighting the increased prevalence of peri-implant diseases.

Smoking has been considered a major risk factor for both tooth loss (Mai et al., 2013; Salvi et al., 2014) and peri-implant diseases (Javed et al., 2019; Rokaya et al., 2020; Romandini et al., 2021). It has also been reported to impact negatively on the outcomes of peri-implant treatments (Konstantinidis et al., 2015; Schwarz et al., 2018; AlJasser et al., 2021). However, these impacts can go undetectable until the emergence of clinical signs. There is a very limited number of studies investigating the association between smoking-induced microbial alterations and poor responsiveness to peri-implant treatments in smokers. Considering the biofilm-mediated infectious nature of peri-implant diseases, we hypothesize that the impact of smoking can be reflected on the peri-implant microbiome even in status of clinical health, which may increase the stubbornness of the microbial community, compromising the outcomes of peri-implant treatments.

Existing studies in this field using high-throughput sequencing have shown that smoking is associated with changes in the relative abundance of certain health- or disease-associated species, as well as alterations in the diversity of the peri-implant microbiome (Tsigarida et al., 2015; Pimentel et al., 2018). These studies mainly focused on taxonomical or functional aspects. Local stability, as described in former studies (Allesina and Tang, 2012; Coyte et al., 2015), measures the capability of a microbial community to return to its former equilibrium within an infinitesimal time interval after being subjected to minute perturbations. In a more macroscopic view, a community with higher local stability is less likely to shift from one status to another. Explorations on how smoking influences the community structure and local stability of the peri-implant microbiome are very limited. To test our hypothesis, the present study is aimed to compare the structural properties of peri-implant microbial communities between smokers and non-smokers through a cross-sectional design. By combining ecological dynamic models with high-throughput sequencing data, we aimed to investigate if the peri-implant microbiome associated with smoking has greater local microbial stability compared to non-smoking group. Furthermore, we also investigated if there are smoking-associated alterations in the topological structures of microbial community which may compromise the responsiveness towards peri-implant treatments in smokers even in status of peri-implant health.

## Materials and methods

### Participant recruitment

This study was approved by the Ethics Committee of the College of Stomatology, Xi’an Jiaotong University (xjkqll [2020] NO.016). All recruited participants provided written consent.

After screening through 231 patients who had received dental implants to replace missing teeth at College of Stomatology, Xi’an Jiaotong University, 18 participants including 10 current-smokers (S) and 8 non-smokers (those who had never smoked, NS), with clinically healthy implants were enrolled in this study according to our inclusion and exclusion criteria (**Table 1**). The clinical and demographical characteristics were recorded (**Table 2**). Clinical assessment of the implants was conducted according to the consensus report of the Sixth European Workshop on Periodontology (Lindhe et al., 2008). All measurements and sampling procedures were performed by the same clinician. Before the study, intra-examiner calibration was performed and the examiner was evaluated as consistent throughout the calibration.

**Table 1:** Detailed inclusion and exclusion criteria.

	The inclusion criteria	The exclusion criteria
	A single implant with a cement-retained crown in function for over 2 years No presence of redness, suppuration, bleeding on probing in the surrounding soft tissue Radiographical marginal bone loss of less than 2 mm compared to baseline	History of periodontitis Diabetes mellitus or other severe systemic diseases HIV infection or other severe immune diseases or history of immunosuppressant therapy History of bisphosphonates, steroids, or other therapy influencing bone metabolism Antibiotic therapy, oral antiseptic therapy, or oral prophylactic treatment in the past 3 months Other dentures in any form Pregnancy or lactation Age over 60 years
S group	Current smokers with a 10-pack-year or greater history of tobacco usage	
NS Group	No history of tobacco usage	

**Table 2:** Clinical and demographical characteristics between Smoker (S) and Non-Smoker (NS) Group.

	S Group	NS Group	*p value*
Age (mean ± SD, y)	51.10 ± 10.16	45.86 ± 10.91	0.167
Gender			
Female	1	3	
Male	9	5	
Smoking history (mean ± SD, pack-year)	27 ± 12.29	0 ± 0	
Probing depth (mean ± SD, mm)	2.80 ± 0.63	2.88 ± 0.83	0.884
Width of keratinized gingiva* (mean ± SD, mm)	4.10 ± 0.99	4.25 ± 1.04	0.744
Radiographical bone loss (mean ± SD, mm)	0.96 ± 0.36	1.22 ± 0.37	0.130
Periodontal condition of adjacent teeth (n)			
Health	8	7	
Gingivitis	2	1	
Years in function mean ± SD, mm	4.80 ± 1.62	4.50 ± 1.69	0.714
Implant location (n)			
Anterior	2	1	
Posterior	8	7	
Implant type (n)			
Bego (RS, SC)	9	8	
Osstem (TS III)	1	0	

*The width of the keratinized gingiva was measured on the buccal or labial side of the implant. Statistical comparison was carried out using Mann–Whitney U test.

The examiner was asked to measure the probing depth (PD), bleeding over probing (BOP), and marginal bone loss (MBL) of 40 implants that were not included in this study. Each implant was measured 3 times with a minimum interval of 1 hour within the same day. The results for PD and MBL were taken as consistent if the error among the three measurements was no more than 1mm, while the results for BOP were considered consistent when all three measurements had the same results (positive or negative). The percentage of consistency for PD, MBL, BOP was 87.5%, 92.5%, and 90.0% respectively.

### Sample collection

The participant was first asked to rinse the mouth with distilled water. The implant was then isolated using cotton rolls. The supragingival plaques were carefully removed with sterile scalers and cotton swabs (Hu-Friedy, Chicago, USA). Sterile endodontic paper points (Gapadent, Tianjin, China) were then used to collect the subgingival peri-implant biofilms by gently inserting the paper points as deep as possible into the peri-implant sulcus and leaving them for 20 seconds (Nickles et al., 2017). After removal, the paper points were placed in 1.5 ml microcentrifuge tubes (Biosharp, Beijing, China) containing phosphate-buffered saline (Biosharp, Beijing, China). The above process was performed with particular caution to avoid contamination from blood, saliva, and tooth surfaces. For each participant, 4 samples were collected from the buccal, lingual, mesial, and distal sulcus of the implant. The samples were frozen at -80°C and then transported to Personal Biotechnology Co., Ltd. (PersonalBio, Shanghai, China) *via* cold chain for further procedures.

### DNA isolation and metagenomic sequencing

The genomic DNA of the samples was extracted following the hexadecyltrimethylammonium bromide (CTAB) protocol (Wilson, 2001). After DNA extraction the 4 samples from the same participant were pooled. The quantity of the extracted DNA was measured using a NanoDrop ND-1000 spectrophotometer (Thermo Fisher Scientific, Waltham, USA). Agarose gel electrophoresis was then performed to evaluate the quality of the acquired DNA. An Illumina TruSeq Nano DNA LT Library Preparation Kit was then used to construct the genomic libraries for shotgun sequencing. The prepared libraries were sequenced by an Illumina HiSeq X-ten platform (Illumina, USA).

### Metagenomic analysis

#### Data filtration and host data removal

With prinseq++ (Schmieder and Edwards, 2011), we filtered the raw reads when they contained more than 20% low-quality bases (<Q20) or 15 bases of adapter sequences. For the paired-end reads, we discarded the read pairs if either end was recognized as low quality. Then, we aligned the filtered sequencing data to the human genome (hg38) using Bowtie2 (v2.4.4) (Langmead and Salzberg, 2012), removed the reads whose alignment length exceeded forty percent of the read length, and kept the filtered data for further analysis.

#### Taxonomical and functional annotation

Applying MetaPhlAn3 (version 3.0.7) and microbe marker genes (mpa_v30_CHOCOPhlAn_201901) (Beghini et al., 2021), we obtained the taxonomical assignments and abundance information for all samples. Using HUMANN3 software (version 3.0), we detected the distributions of bacterial functions for the oral microbiome and visualized the specific functional categories with GraPhlan (Asnicar et al., 2015).

#### PCoA analysis

Based on the Bray-Curtis distances, we performed principal coordinate analysis (PCoA) for all samples by adopting the package “vegan” in R and plotted the results by using the package “ggplot2” in R.

#### GM network construction and characterization

With the species profiles in each group, we calculated the Spearman correlation coefficients among the species by using the package “psych” in R and kept the relations with coefficients< -0.6 or > 0.6 (P< 0.05). Then, we plotted the GM co-occurrence networks by applying Gephi (Version 0.9.2) (Bastian et al., 2009).

Modularity, eigenvector centralization, as well as clustering coefficient are important indicators for describing the topological structure of complex networks (Watts and Strogatz, 1998; Grilli et al., 2016; Bienenstock and Bonacich, 2021). Modularity is the extent to which a system’s components may be divided into small, intra-connected groups called modules. Eigenvector centralization measures the assortativity of the high-degree species, or in other words how frequently and closely the high-degree species connected with each other. In contrast, the clustering coefficient describes the neighborship of all the species in the network, regardless of their degrees. To characterize these topological features of the GM co-occurrence networks, we adopted the package “igraph” in R and analyzed the topological properties of each network.

#### Statistical analysis

With the species profiling obtained from MetaPhlAn3 software (Beghini et al., 2021), we assessed the α-diversity using the Shannon index (package “vegan” in R). The study adopted the Mann-Whitney u test to compare the differences in clinical and demographical characteristics. For the comparison of species and functional abundances among the groups, the Wilcoxon rank–sum test was adopted, and the results were adjusted with the Benjamini and Hochberg method (FDR< 0.05) using “p.adjust” in R.

### Local stability analysis

In a previous study (Zhang et al., 2021), we introduced a strategy to predict and compare the stability differences between microbial communities from cross-sectional sequencing data following studies by May et al. and Allesina et al. (May, 1972; May, 1973; Allesina and Tang, 2012). Briefly, if we impose a sufficiently small perturbation on a microbial community resting at equilibrium, the evolution of the perturbation can be approximated by the following equation:


$$\frac{dx(t)}{dt}\approx Mx(t)$$

where vector *x*(*t*) describes the deviation from the equilibrium abundance, *M* is the so-called community matrix, whose diagonal elements (i.e., *M*_*ii*_ refer to the self-regulating effect of species *i* for preventing itself from growing beyond the limit that the environmental resources can feed and whose off-diagonal elements (i.e., *M*_*ij*_ are the effects of species *j* on species *i* Mathematically, a community is stable if all the eigenvalues of *M* have negative real parts. Therefore, the real part of the rightmost eigenvalue can be used to quantify the stability of a microbial community.

The community matrix *M* was constructed by the following steps. First, we extracted the adjacency matrix *K* , whose elements *K*_*j*_ are the Spearman correlation coefficients between the abundances of species *i* and *j* , which was extracted from our taxonomic data. Then, based on *K* , we set the elements of *M* as:


$$\{\begin{array}{c} K_{ij}>0.6\to M_{ij}=\left|X\right|\\ K_{ij}<-0.6\to M_{ij}=-\left|X\right|\\ M_{ii}=-1 \end{array}$$

where *X* is a random variable following a normal distribution *N*(*μ*,*σ* ^2^) . By changing the values of *μ* and *σ* , we performed a series of simulations to calculate the eigenvalues of *M* to compare the stability between different microbial communities.

## Results

### Taxonomical and functional features of the peri-implant microbiome

**Figure 1:**
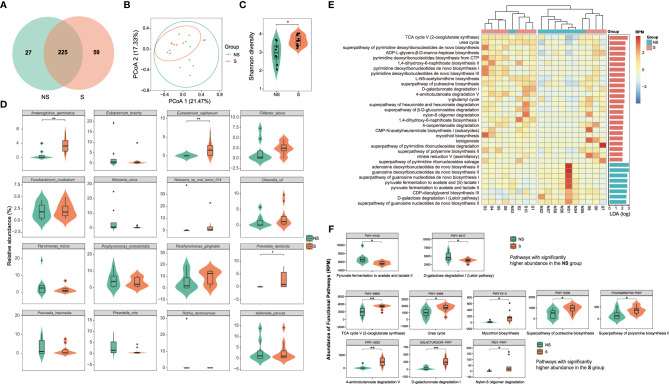
**(A)** Venn diagram showing the number of shared and exclusive species in the smoking (S) and non-smoking (NS) groups. **(B)** Beta diversity analysis using PCoA. A clear clustering tendency was observed in the smoking group, indicating higher homogeneity of bacterial profiles among smokers. **(C)** Alpha diversity analysis based on the Shannon index. The Shannon index of the smoking microbiome was significantly higher than that of the nonsmoking microbiome (p < 0.05, Wilcoxon rank-sum test), indicating that a higher alpha diversity was associated with smoking. **(D)** The relative abundances of the core species are visualized using violin plots. *Anaeroglobus geminatus*, *Eubacterium saphenum*, and *Prevotella denticola* showed significantly higher abundances among smokers (p < 0.05, Wilcoxon rank-sum test). **(E)** Heatmap presenting the gene abundances of all 33 differentiating functional pathways based on the reads number per million reads (RPM). The samples were classified based on hierarchical clustering. Labels on the right show the score from linear discriminant analysis (LDA). **(F)** Detailed RPM of the functional pathways with significantly different abundance between the two groups are visualized using violin plots. * for p < 0.05; ** for p < 0.01.

A total of 299,210,888 paired-end (PE) reads were obtained from our samples, with an average of 16,622,827.11 ± 9,118,377.35 (mean ± SD) PE reads per sample (ranging from 4,641,386 to 32,133,756). After alignment to the database, a total of 311 bacterial species were identified in the peri-implant sulcus (**Figure 1A**). Differences in the beta diversity, alpha diversity, and abundance of the core microbiome (species shared by at least 80% of individuals with a minimum relative abundance of 0.1%) indicated distinct microbial composition between the S and NS groups (**Figures 1B–D** and **Data Sheet 1**). The differences associated with smoking were also reflected in the aspect of genomic functions. Out of 403 functions identified in the clean reads, 33 pathways showed significantly different gene abundances (**Figure 1E** and **Data Sheet 2**). Besides the differential functions related to ribonucleotide and deoxyribonucleotide metabolism, we also visualized the detailed gene abundance of those functions characterizing the S and NS groups (**Figure 1F**). These findings indicate that the peri-implant microbiomes from smokers and non-smokers were distinct in both taxonomical and functional aspects.

### The microbial community of smokers was more stubborn in terms of local stability

**Figure 2:**
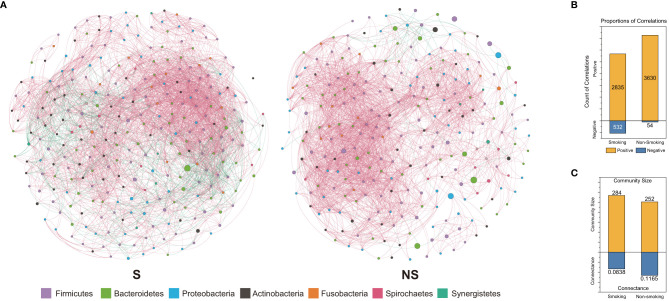
**(A)** Bacterial co-occurrence networks of smoking and nonsmoking groups. Each node in the network represents a bacterial species. The colour of the node indicates the phylum of the species, while the size reflects its abundance. Edges linking nodes represent established correlations- between species. Red edges represent positive correlations, while green edges represent negative correlations. **(B)** Bar charts showing the count and proportion of positive/negative correlations in the smoking and nonsmoking groups. **(C)** Bar charts showing the community size and total connectance in the smoking and nonsmoking groups.

**Figure 3:**
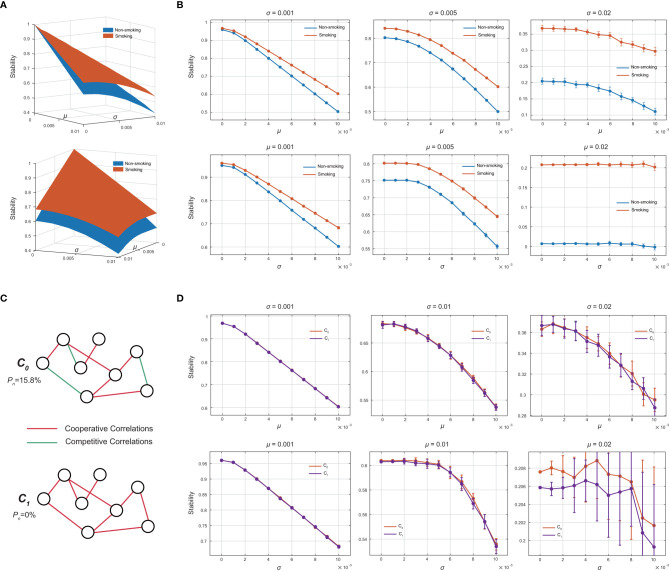
**(A)** The strengths of the bacterial interactions were set to follow a normal distribution *N*(*μ*,*σ* ^2^) . By changing the values of *μ* and *σ* (horizontal axes), the stability of the smoking and nonsmoking microbiomes (vertical axis) is represented by two curves. Two perspectives are shown here. **(B)** Sections from the three-dimensional curves in (a). Each dot on the line represents the mean stability value of 50 simulation repeats. The error bar shows the standard deviation of these repeats. The plot shows a clear tendency for higher stability in the smoking microbiome than in the nonsmoking microbiome under various parameter settings. **(C)** A schematic diagram showing the difference between C_0_ and C_1_. C_0_ is the original community from the smoking microbiome in our study, with a proportion of negative correlations (*P_n_*) of 15.8%. C_1_ is the virtual community modified from C_0_ by changing all the negative correlations into positive ones. **(D)** The stability difference between C_0_ and C_1_ is also visualized. The two curves are very close and intermingled, indicating similar stability between the original and modified communities.

To compare the local stability between S and NS groups, we first visualized the bacterial co-occurrence networks of both groups together with the basic properties of these networks (**Figure 2**). Dynamic simulations were performed to compare the local stability between the smoking and non-smoking groups from our cross-sectional samples (**Figures 3A, B**). Briefly, the interactions between species were inferred from the co-occurrence networks. Then by assigning the strength of these interactions to a normal distribution under various parameter settings, we were able to capture the tendency of how the local stability differed between the S and NS groups (see *Materials and methods*). These analyses revealed a clear tendency for the peri-implant microbial community of the smoking group to have higher local stability than the nonsmoking group. The same conclusion could be drawn under numerous parameter settings, proving the robustness of our finding.

The higher local stability in the smoking group indicates that the peri-implant microbiome had become more stubborn under smoking conditions and could not be shifted easily when subject to exogenous perturbations. This may be related to the poor responsiveness towards peri-implant treatment in smokers.

### Alterations in the microbial community structures compromised the responsiveness of the peri-implant microbiome

Inter-species correlations, as well as other structural properties have been proven to influence the stability of microbial communities (Gardner and Ashby, 1970; Allesina and Tang, 2012; Coyte et al., 2015). The combined effect of alterations in both correlation types and structural properties together determined the high stability in the smoking-related microbiome. However, we were also interested in determining which of the two factors was the greater contributor to the difference in local stability, and therefore finding out what in specific had led to the greater stubbornness and the compromised responsiveness of peri-implant microbiome in smokers.

To evaluate the association between the percentage of negative/positive correlations and the local stability of the peri-implant microbiome, we constructed a virtual microbial community by changing all the negative correlations in the smoking network into positive correlations. This community was named C_1_ as a counterpart to its original smoking microbiome C_0_ (**Figure 3C**). In this way, C_1_ and C_0_ had exactly the same community size, connectance, and degree distribution; the only difference was that the proportion of negative correlations (*P_n_*) in C_0_ was 15.8% versus 0 in C_1_. The stability difference between C_1_ and C_0_ were then compared using the same strategy above to evaluate the stability contribution from negative correlations (**Figure 3D**). The results showed that C_1_ and C_0_ had almost the same local stability under various parameter settings, which indicates that the percentage of negative/positive correlations was not essential in determining the local stability of the peri-implant microbiome.

**Figure 4:**
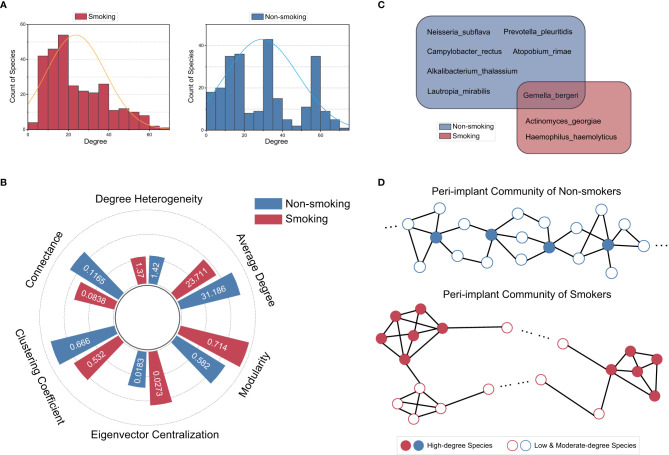
**(A)** Degree distribution of the smoking and nonsmoking communities. The fitting curves of both groups demonstrate a right-skewed tendency. Especially in the smoking group, the number of high-degree species is very limited. **(B)** Analysis of topological properties. Smoking is associated with higher modularity and eigenvector centralization, and is also associated with lower clustering coefficient and density in the correlation network. **(C)** The articulation species in the two groups are shown in the Venn diagram. Articulation species referred to those bridging connectors between modules. **(D)** Based on the topological properties acquired, schematic diagrams are plotted to show the different patterns of how the species were connected in the two groups. In the nonsmoking group, all species are connected more proportionately than in the smoking group. The whole network is more evenly clustered, and there are more articulation species bridging the network. In the smoking group, high-degree species preferentially interact with each other, dividing the network into several modules. There are also fewer articulation species and a tendency toward less clustering.

We then visualized more structural properties of the peri-implant microbiome to further explore their impact on the local stability. The degree distributions showed that there were more species in lower- or moderate-degree regions than there were in the high-degree region, especially in the S group (**Figure 4A**). Although the degree heterogeneity (**Figure 4B**) indicated that the extent of deviation from a completely homogeneous distribution was similar in the two groups, the specific degree of each species changed extensively (see **Data Sheet 3**), implying that the structure of the peri-implant microbiome was poorly conserved under the influence of smoking. This was confirmed by other topological analyses.

The topological properties including Modularity, eigenvector centralization, as well as clustering coefficient of the S and NS networks were shown in **Figure 4B**. The network of the smoking microbiome demonstrated higher eigenvector centralization and higher modularity. This indicated that the minority high-degree species in the smoking network preferentially connected with one another (Negre et al., 2018), dividing the community into some “small world” modules within certain groups of species. This consequently weakened the spread of any signal (e.g., alterations in species abundance) that was input to the remaining, majority lower-degree species, as signals transmitted less efficiently between modules than within them (Bienenstock and Bonacich, 2021). However, in the nonsmoking group, the correlations among the species were more proportionate, and the spread of input signals was facilitated as the network was less modularized. The nonsmoking network also contained more articulating species (connectors between modules) than the smoking group (**Figure 4C**). These species played essential roles in the transmission of the signals by linking different modules.

The above findings suggested that under the influence of smoking, the chain effect consequent to changes in the abundances of some species was hampered and spread more slowly to other species without the bridging effect of these articulating species. According to these properties, schematic diagrams of the peri-implant community structure from smokers and non-smokers were plotted (**Figure 4D**). The structural differences showed that the microbial communities in the smoking group had more resilience against exogenous perturbations than those in the nonsmoking group, and explained why the peri-implant microbiome became less responsive to abundance-altering therapies such as subgingival scaling or local usage of antibiotics under the influence of smoking.

## Discussion

Within the limit of the present study, the primary finding was that in addition to the aberrant changes in taxonomical and functional aspects, smoking also extensively altered the community structure and increased the stubbornness of the peri-implant microbiome even in status of clinical health. This suggests that these structural alterations potentially make the microbiome in smokers less responsive towards exogenous perturbations.

Smoking is known to affect the composition and metabolism of the oral microbiome and is considered a major risk factor for peri-implantitis or periodontitis (Bizzarro et al., 2013; Moon et al., 2015; Tsigarida et al., 2015; Duan et al., 2017; Pimentel et al., 2018; Torrungruang et al., 2020; Amerio et al., 2022). Our results are consistent with previous studies, showing that even for a clinically healthy peri-implant environment, smoking increases the abundances of putative pathogens such as *A. geminatus*, *E. saphenum*, and *P. denticola* (Abusleme et al., 2013; Bao et al., 2017; Al Kawas et al., 2021) and regulates the functional potential of the peri-implant microbiome (**Figure 1**), shifting it to a more disease-related, sub-healthy status (Tsigarida et al., 2015).

In addition, the influence of smoking was also accompanied by extinction-colonization dynamics. As certain species were either lost or gained, the overall structure of the peri-implant community was poorly conserved. Furthermore, the high local stability of the smoking microbiome (**Figures 3A, B**) highlights that smoking alters the peri-implant microbiome into a more stubborn state and compromises the responsiveness of the microbiome, which negatively effects the outcome of the treatments (e.g., subgingival scaling or local usage of antibiotics/antiseptics). This is in agreement with multiple previous studies, both on peri-implant and periodontal diseases (Jin et al., 2000; Labriola et al., 2000; Konstantinidis et al., 2015; Alexandridi et al., 2018; Nie et al., 2020; AlJasser et al., 2021).

Our results also suggested that rather than the proportions of negative/positive correlations, it was the topological structure that caused the distinct local stability of the peri-implant microbiome between smokers and non-smokers (**Figure 4B**). Differences in eigenvector centralization, modularity, and clustering coefficient together determined the dissimilarity of the topological patterns between the S and NS groups (Watts and Strogatz, 1998; Serban, 2020; Bienenstock and Bonacich, 2021; Alcala-Corona et al., 2021) (**Figure 4D**).

In the pattern of the smoking microbiome, high-degree species preferentially connected with one another and formed several “small world” modules. The few articulation species acted as connectors between modules. The network was overall less clustered because the modules were separated by many low-degree species. The property of this pattern is that signals from the high-degree region can be amplified due to the high assortativity of these species (Bienenstock and Bonacich, 2021). However, signals from the low-degree region can be difficult to spread through the whole network because of the distant relationships between modules.

In the pattern of the nonsmoking microbiome, the connections between high-degree and low-degree species were more proportionate. The whole network was less modularized, and there were more articulation species bridging the high-degree species, making the network overall more evenly distributed. In this pattern, the signals can spread more easily and smoothly, whether they started on high-degree species or low-degree species.

Suppose that an exogenous signal is applied randomly to some species within the peri-implant microbiome. This signal can be any perturbation that alters the abundance of the species, such as a peri-implant therapy or routine oral hygiene maintenance. Given the right-skewed degree distributions of both groups (**Figure 4A**), it is easy to understand that this signal could be applied to low- or moderate-degree species with greater probability. In this case, the spread of the signal will be less easy in the smoking group than in the nonsmoking group, which means that it is more difficult to alter the microbial communities in smokers. This is where the smoking microbiome gains its exceptional local stability and may be the reason why smokers are less responsive to periodontal and peri-implant therapies from a microbial perspective.

To reverse this tendency, we suggest that later studies may focus on modifying the community structure of the peri-implant microbiome in smokers. One possible option is to introduce some articulating species into the microbial community. In this study, the species exclusive to the NS group are *Neisseria subflava*, *Prevotella pleuritidis*, *Campylobacter rectus*, *Atopobium rimae*, *Alkalibacterium thalassium*, and *Lautropia mirabilis*. Introducing these species into the smoking microbiome can theoretically downregulate the extent of modularity and centralization of the peri-implant microbiome, and may help promote the effect of related therapies. This embraces a similar concept to the probiotics therapy. Nonetheless, there is currently a scarcity of evidence that can substantiate the usage of probiotics in treating peri-implant or periodontal diseases (Barootchi et al., 2020; Gao et al., 2020; Zhao et al., 2020; Ng et al., 2021). Our hypothesis is that utilizing a single species of conventional probiotics like *Lactobacillus reuteri* or *Lactobacillus salivarius* (Meurman and Stamatova, 2007) may not be sufficient enough to impact the whole microbial community, especially when they are, in fact, not the articulation points in the peri-implant microbiome. However, our hypothesis was mainly based on mathematical derivations and dynamic simulations. To further validate this, and to further ascertain what species are the most efficient in modifying the peri-implant community, the experimental evidences will also be needed.

Another major limitation that must be mentioned is the sample size of this work, which was small to represent the whole population. Limited sample size may have precluded us from extracting precise correlations among species. But the overall tendency of alterations was similar with previous studies (Jin et al., 2000; Labriola et al., 2000; Konstantinidis et al., 2015; Tsigarida et al., 2015; Pimentel et al., 2018; Alexandridi et al., 2018; Nie et al., 2020; AlJasser et al., 2021), and also coincided with our previous work using a greater dataset (Zhang et al., 2021). In addition, we performed statistical analysis where applicable to ensure the reliability of our results, and hope that our methods and results may provide foundation for further exploring how smoking impact the peri-implant microbiome and also on developing new strategies in improving the outcome of peri-implant treatment in smokers. However, to provide more powerful and meaningful implications, we suggest that future studies increase the sample size or integrate pre-existing datasets to verify the generalizability of our findings.

## Data availability statement

The data presented in the study are deposited in the NCBI BioProject repository, accession number PRJNA837034.

## Ethics statement

The studies involving human participants were reviewed and approved by Ethics Committee of the College of Stomatology, Xi’an Jiaotong University. The patients/participants provided their written informed consent to participate in this study. Written informed consent was obtained from the individual(s) for the publication of any potentially identifiable images or data included in this article.

## Author contributions

YZ: Designed the study; collected the samples; analysed the data; performed the statistical analyses; drafted and revised the manuscript. SN: Interpreted the results, drafted and revised the manuscript. YY: Designed the study; analyzed the data; performed dynamic simulations; revised the manuscript. YW: analysed the data; revised the manuscript. XC: performed the statistical analyses; revised the manuscript. YBL: collected the samples; revised the manuscript. YHL: Designed the study; analysed the data; performed statistical analyses; revised the manuscript. QZ: Supervised the program; raised the funding; designed the study; revised the manuscript. All authors contributed to the article and approved the submitted version.

## Funding

This study was partly funded by Key Research and Development Program of Shaanxi Province, China (program code 2019SF-144).

## Acknowledgments

We would like to thank all involved patients for their cooperation. We would specially like to thank Prof. Shengbin Li of Bio-evidence Sciences Academy, Xi’an Jiaotong University for his valuable advice on designing and conducting this study. We would also like to thank all nurses and clinicians in the Department of Implant Dentistry, College of Stomatology, Xi’an Jiaotong University for their assistance in sample collection and clinical examination.

## Conflict of interest

The authors declare that the research was conducted in the absence of any commercial or financial relationships that could be construed as a potential conflict of interest.

## Publisher’s note

All claims expressed in this article are solely those of the authors and do not necessarily represent those of their affiliated organizations, or those of the publisher, the editors and the reviewers. Any product that may be evaluated in this article, or claim that may be made by its manufacturer, is not guaranteed or endorsed by the publisher.
